# Effects of tryptophan-rich breakfast and light exposure during the daytime on melatonin secretion at night

**DOI:** 10.1186/1880-6805-33-33

**Published:** 2014-11-19

**Authors:** Haruna Fukushige, Yumi Fukuda, Mizuho Tanaka, Kaoru Inami, Kai Wada, Yuki Tsumura, Masayuki Kondo, Tetsuo Harada, Tomoko Wakamura, Takeshi Morita

**Affiliations:** Department of Human Health Sciences, Graduate School of Medicine, Kyoto University, 53, Shogoin, Kawahara-cho, Sakyo-ku, Kyoto 606-8507 Japan; Department of Environmental Science, Fukuoka Women’s University, 1-1-1, Kasumigaoka, Higashi-ku, Fukuoka, 813-8529 Japan; Department of Living Environmental Science, Fukuoka Women’s University, 1-1-1, Kasumigaoka, Higashi-ku, Fukuoka, 813-8529 Japan; Laboratory of Environmental Physiology, Graduate School of Integrated Arts and Sciences, Kochi University, 2-5-1, Akebonocho, Kochi, 780-8520 Japan; Department of Food and Nutrition, Junshin Junior College, 1-1-1, Chikushigaoka, Minami-ku, Fukuoka 815-8510 Japan; Comprehensive Housing R & D Institute, Sekisui House Ltd, 6-6-4, Kabutodai, Kizugawa-city, Kyoto 619-0224 Japan

**Keywords:** Bright light exposure, Circadian rhythm, Melatonin, Sleep, Tryptophan

## Abstract

**Background:**

The purpose of the present study is to investigate effects of tryptophan intake and light exposure on melatonin secretion and sleep by modifying tryptophan ingestion at breakfast and light exposure during the daytime, and measuring sleep quality (by using actigraphy and the OSA sleep inventory) and melatonin secretion at night.

**Methods:**

Thirty three male University students (mean ± SD age: 22 ± 3.1 years) completed the experiments lasting 5 days and 4 nights. The subjects were randomly divided into four groups: Poor*Dim (n = 10), meaning a tryptophan-poor breakfast (55 mg/meal) in the morning and dim light environment (<50 lx) during the daytime; Rich*Dim (n = 7), tryptophan-rich breakfast (476 mg/meal) and dim light environment; Poor*Bright (n = 9), tryptophan-poor breakfast and bright light environment (>5,000 lx); and Rich*Bright (n = 7), tryptophan-rich breakfast and bright light.

**Results:**

Saliva melatonin concentrations on the fourth day were significantly lower than on the first day in the Poor*Dim group, whereas they were higher on the fourth day in the Rich*Bright group. Creatinine-adjusted melatonin in urine showed the same direction as saliva melatonin concentrations. These results indicate that the combination of a tryptophan-rich breakfast and bright light exposure during the daytime could promote melatonin secretion at night; further, the observations that the Rich*Bright group had higher melatonin concentrations than the Rich*Dim group, despite no significant differences being observed between the Poor*Dim and Rich*Dim groups nor the Poor*Bright and Rich*Bright groups, suggest that bright light exposure in the daytime is an important contributor to raised melatonin levels in the evening.

**Conclusions:**

This study is the first to report the quantitative effects of changed tryptophan intake at breakfast combined with daytime light exposure on melatonin secretion and sleep quality. Evening saliva melatonin secretion changed significantly and indicated that a tryptophan-rich breakfast and bright light exposure during the daytime promoted melatonin secretion at this time.

## Background

Melatonin is a hormone secreted by the pineal gland in the brain. Its secretion is controlled by the suprachiasmatic nucleus in the hypothalamus, the central biological clock of circadian rhythms in humans. Daily exposure to light resets the phase of the clock in the suprachiasmatic nucleus [1]. Melatonin secretion usually shows a peak at midnight, and it is often used as an indicator of sleep quality and phase advances or delays of the sleep-wake cycle [2, 3]. In the clinical field, clinical doctors and researchers use melatonin to improve sleep quality: to treat insomnia and depression and to reduce jet lag, and it is recommended by some clinicians as a preventive agent for breast cancer [4–9].

Tryptophan is one of the essential amino acids and is contained in proteins sourced from milk, eggs, meat, grains, and beans. It can cross the blood–brain barrier and is transformed into serotonin in the brain, subsequently converted to melatonin. The biosynthesis of melatonin is rate-limited by the activity of arylalkylamine N-acetyltransferase [10]. Zawilska et al. reported that arylalkylamine N-acetyltransferase activity is affected by light; its activity declines in the photoperiod (while melatonin secretion also decreases) and increases in the scotoperiod (when melatonin secretion also increases) [11]. Therefore, tryptophan intake and the timing of light exposure must be considered together if the effect of tryptophan upon melatonin secretion and sleep quality is to be maximized.

Hudson et al. [12] reported that using protein as a source of tryptophan before sleep improved its quality at night by amounts that were comparable with the intake of pharmaceutical-grade tryptophan. Markus et al. [13] examined the effects of tryptophan intake (from different sources) at breakfast on the plasma tryptophan/large neutral amino acids (TRP/LNAA) ratio and mood. The results showed that intake of a tryptophan-rich source from hydrolyzed protein had significantly greater effects on the plasma TRP/LNAA ratio and improvement of mood than did pure tryptophan and alpha-lactalbumin whey protein. In addition, Markus et al. [14] measured depressive mood and perceptual-motor and vigilance performance in subjects under stress (who had high or low chronic stress vulnerabilities) after they had ingested tryptophan-rich egg protein hydrolysate in the morning. The results revealed that egg protein hydrolysate improved the depressive mood in all subjects and perceptual-motor and vigilance performance in those subjects who had low chronic stress vulnerability. These experiments were performed with the aim of examining the effects of tryptophan intake in the morning on serotonin synthesis, which improves mood and mental activity. However, an important factor for this aspect of tryptophan function – light intensity during the daytime – was not considered, and effects on sleep and melatonin secretion were not investigated.

Nakade et al. [15] reported the relationship between tryptophan intake and sleep quality (in children aged 2 to 6) in a survey of daily breakfast composition, morning light exposure, and sleep. They found that tryptophan intake at breakfast, coupled with morning light exposure, was associated with higher melatonin secretion and easier onset of sleep the following night. There is also evidence that tryptophan intake in the morning and light exposure at night affect the following sleep; Wada et al. [16] reported that tryptophan-rich breakfasts and low-color temperature light sources at night increased saliva melatonin concentration in University soccer club members. Nevertheless, these investigations were carried out without regulation of several aspects of the individuals’ lifestyle, including overall diet and daytime light exposure.

The purpose of the present study is to investigate effects of tryptophan intake and light exposure on sleep and melatonin secretion, by modifying tryptophan ingestion at breakfast and light exposure during the daytime, and measuring sleep quality and melatonin secretion at night.

## Methods

### Subjects and group setting

The experiment was performed during September 17th to 21st, 2012 (lasting 5 days and 4 nights) on 40 male University students, none of whom had any mental disorders (Cornell Medical Index), food allergies, or were taking drugs. The data from 33 of them (mean ± SD age: 22 ± 3.1 years, height: 173 ± 5.5 cm, weight: 61.3 ± 6.7 kg, BMI: 20.5 ± 2.3) were analyzed as three subjects withdrew and four subjects’ saliva samples were too small to be analyzed. The subjects were randomly divided into four groups: Poor*Dim (n = 10), tryptophan-poor breakfast (55 mg/meal) in the morning (between 07:30 h and 08:00 h) and dim light environment (<50 lx) during the daytime (between 07:30 h and 18:00 h); Rich*Dim (n = 7), tryptophan-rich breakfast (476 mg/meal) and dim light environment; Poor*Bright (n = 9), tryptophan-poor breakfast and bright light environment (>5,000 lx); and Rich*Bright (n = 7), tryptophan-rich breakfast and bright light environment. It is more convenient and practical for healthy young people living a normal, busy lifestyle if the tryptophan in their diet is derived from common foodstuffs; therefore, the tryptophan-poor breakfast consisted mainly of vegetables, such as salad and orange juice, whereas the tryptophan-rich breakfast was loaded with proteins from food sources such as salmon and *natto* (fermented soybeans; a Japanese traditional food) rather than from nutritional supplements. The experimental conditions and subjects’ physical characteristics for the four groups are shown in Table 1. Subjects’ age, height, weight, and BMI were not significantly different between the four groups (one-way ANOVA). Table 2 shows the estimated intake of energy and basic nutrients, including tryptophan, at breakfast, lunch, and supper. The breakfast menus were designed to differ in tryptophan content (TRP-Poor and TRP-Rich breakfasts) but have minimal changes in other nutrients and energy. For each group, breakfast, lunch, and supper were the same on all experiment days. The energy and nutrient intakes of the controlled food were calculated according to the Standard Tables of Food Composition in Japan (5th Revised and Enlarged Edition). Lunch and supper were controlled with contents that were recommended as appropriate for healthy young males by the Ministry of Health, Labour and Welfare of Japan.

**Table 1:** **The four experimental groups**

Group	Poor*Dim	Rich*Dim	Poor*Bright	Rich*Bright
Number of subjects	10	7	9	7
Age (years)*^1^	22.1 (4.6)	21.4 (2.5)	21.0 (2.4)	21.4 (2.1)
Height (cm)*^1^	170.9 (6.1)	175.1 (5.1)	173.1 (4.0)	174.3 (6.3)
Weight (kg)*^1^	57.7 (5.6)	62.1 (7.4)	64.1 (7.1)	62.0 (5.8)
BMI*^1^	19.9 (2.3)	20.3 (2.9)	21.2 (1.9)	20.6 (2.5)
Tryptophan content of breakfast	Poor*^2^	Rich*^3^	Poor*^2^	Rich*^3^
Lighting condition during the daytime	Dim*^4^	Dim*^4^	Bright*^5^	Bright*^5^

*^1^Mean (SD) is shown for age, height, weight, and BMI.

*^2^Tryptophan content of 55 mg.

*^3^Tryptophan content of 476 mg.

*^4^< 50 lx between 07:30 h and 18:00 h.

*^5^> 5,000 lx between 07:30 h and 18:00 h.

**Table 2:** **Energy and nutrient intake at breakfast, lunch, and supper**

Energy and nutrients		Breakfast		Lunch	Supper
		TRP-Poor	TRP-Rich		
Energy*^1^	(kcal)	378	579	810	723
Tryptophan (TRP)	(mg)	55	476	285	318
Protein	(g)	5.6	36.2	25.6	27.4
Lipid	(g)	7.7	17.5	23.1	16.4
Carbohydrate	(g)	71.7	65.2	121.6	113.9
Sodium	(mg)	813	1118	1742	941
Vitamin B6	(mg)	0.22	0.61	0.49	0.7
Isoleucin	(mg)	162	1649	1021	1084
Leucin	(mg)	311	2855	1826	1964
Phenylalanine	(mg)	200	1731	1028	1096
Tyrosine	(mg)	149	1388	774	898
Valine	(mg)	223	1936	1177	1298
LNAA*^2^	(mg)	1045	9559	5826	6340
TRP/LNAA	(mol/mol)	0.036	0.034	0.033	0.034

*^1^All energy and nutrient intakes of the controlled food in the experiment were calculated according to the Standard Tables of Food Composition in Japan, and the coefficients for each food constituent were different from Atwater’s coefficients.

*^2^LNAA: Large, neutral amino acids (isoleucin, leucin, phenylalanine, tyrosine, and valine).

The experimental protocol was conducted in accordance with the Declaration of Helsinki and conformed to international ethical standards, and was approved by the Ethics Committee at Fukuoka Women’s University. Subjects gave prior written, informed consent and appropriate compensation was given to them after they had completed the experiment.

### Experimental procedure

**Figure 1:**
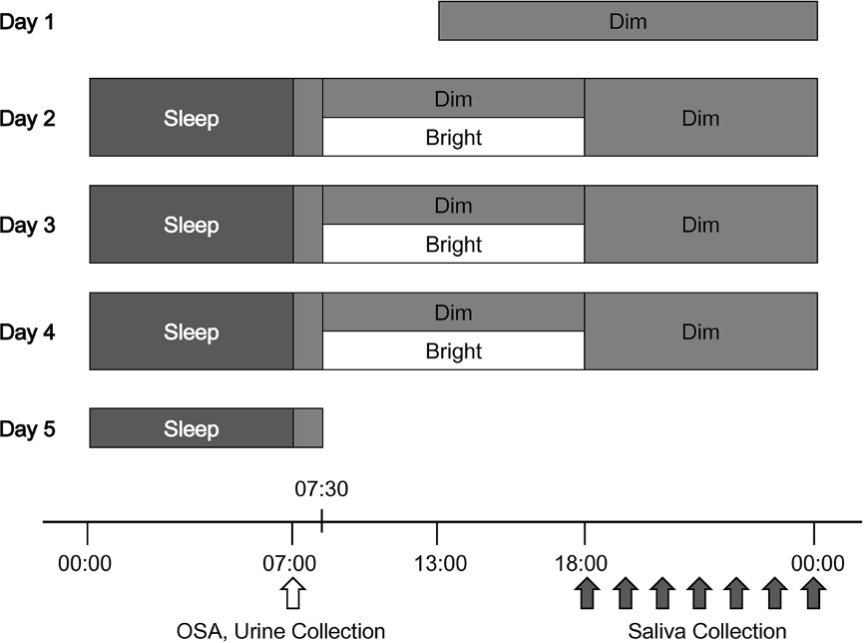
**Experimental protocol.** Breakfast was served between 07:30 h and 08:00 h, lunch between 12:10 h and 12:40 h, and supper between 19:10 h and 19:40 h. For days 2 to 5, subjects rose at 07:00 h and answered the OSAsleep inventory after their urine sample had been collected (indicated by a white arrow underneath the time scale). Subjects in groups Poor*Dim and Rich*Dim continuously stayed under dim light (<50 lx) whereas subjects in groups Poor*Bright and Rich*Bright stayed under bright light (>5,000 lx) between 7:30 h and 18:00 h. Saliva was collected every hour between 18:00 h and 00:00 h (black arrows) and subjects retired at 00:00 h after emptying their bladder.

Figure 1 shows the experimental protocol. For one week prior to the experiment, subjects were asked to eat a vegetable-based breakfast (for tryptophan depletion) and to keep to a regular sleep-wake cycle (retiring at 00:00 h and rising at 07:00 h). Throughout this week, subjects’ adherence to these requirements was checked by objective measurement of activity with an Actiwatch (Mini-Mitter Co. Inc., Oregon, USA) and requiring the subjects to fill in a diary detailing sleep and meals eaten.

The subjects entered individual rooms at 13:00 h on the first day and stayed under a dim light condition (<50 lx, 3,000 K). A curtain was closed and ceiling lights were filtered for the condition. They continuously wore the Actiwatch and their sleep (including sleep efficiency and sleep latency) was monitored throughout the experiment. During days 2 to 4, food and drink intake was regulated (see above), and subjects were required to refrain from watching any video display monitors (cell phone, computer, or TV) in order to regulate light exposure and sensitivity to light. They spent all their time in the chair between 07:00–00:00 h except for using a bathroom. All subjects were allowed to read a book or magazine and/or listen to music, but not to take naps. Breakfast was served between 07:30 h and 08:00 h, lunch between 12:10 h and 12:40 h, and supper between 19:10 h and 19:40 h. Subjects had 200 mL of water with each meal and were free to drink only water between meals.

For days 2 to 5, subjects rose at 07:00 h and answered the OSA sleep inventory [17] after a urine sample had been collected. Subjects in groups Poor*Dim and Rich*Dim remained under dim light between 07:30 h and 18:00 h whereas subjects in groups Poor*Bright and Rich*Bright stayed under bright light (>5,000 lx) with natural sunlight by opening curtain and artificial light by having a lighting device (Bright Light Me, Solartone Ltd., Tokyo, Japan) placed in front of them. Under the bright light condition, natural light changed color temperature accordingly. It was not compulsory for subjects to gaze at the lighting devise. Vertical illuminance and color temperature at eye level were checked for dim and bright conditions by using an illuminometer (Photo Recorder PHR-51, T & D Co., Nagano, Japan) and an illuminance spectrophotometer (CL-500A, Konica Minolta, Inc., Tokyo, Japan) respectively.

Saliva was collected into collection tubes with a pure cotton swab (Sarstedt AG & Co., Tokyo, Japan) every hour between 18:00 h and 00:00 h on days 1 to 4. Saliva samples were immediately centrifuged at 1,500 *g* for 5 min after the collection, and the collection tubes without the cotton swab were then frozen at −30°C until analysis. Subjects retired at 00:00 h after emptying their bladder and their urine was collected between 00:00 h and 07:00 h. The urine samples were also stored at −30°C. Subjects were free to leave after breakfast on day 5. All urinary and salivary analyses were carried out in duplicate and the mean values of the duplicates were used for statistical analyses.

### Analysis method

The following factors were analyzed: i) sleep efficiency and sleep latency from the actigraphs analyzed by the Actiware-Sleep software, as measures of objective sleep quality; ii) subjective estimates of sleep quality on rising, using the OSA sleep inventory (sleepiness, sleep maintenance, worries, integrated sleep feeling, and sleep initiation); and iii) melatonin concentrations in the rising urine sample and saliva samples taken in the 6 hours before retiring. Creatinine-adjusted melatonin, which was not metabolized and was excreted in urine was represented as nighttime melatonin secretion in urine.

Urine samples were analyzed with RIA kits (RK-MEL2 200 tests, Bühlmann Laboratories AG, Schönenbuch, Switzerland). The mean coefficients of variation (CV) of the intra- and inter-assay precision were 7.9% and 11.7%, respectively. The limit of detection (LoD) was 0.3 pg/mL and the limit of quantification (LoQ) was 0.9 pg/mL. Regarding creatinine analysis, Creatinine kits (DeterminerL CRE, Kyowa Medex Co. Ltd., Tokyo, Japan) were used. The mean CVs of the intra- and inter-assay precision were <5% and <3%, respectively. The LoD was 0.04 mg/dL and the LoQ was 0.1 mg/dL. Saliva samples were analyzed with RIA kits (RK-DSM2 200 tests, BÜHLMANN Laboratories AG, Schönenbuch, Switzerland). The mean CVs of the intra- and inter-assay precision were 7.9% and 9.8%, respectively. The LoD was 0.2 pg/mL and the LoQ was 0.9 pg/mL.

The dim light melatonin onset (DLMO) was determined by linear interpolation between adjacent saliva samples using a fixed threshold of 3 pg/mL [18]. For five subjects, their melatonin levels did not rise above the absolute threshold (3 pg/mL) during the sampling period (18:00–00:00 h). Therefore, their data were excluded from DLMO analyses. Phase shift of saliva melatonin secretions was considered by the difference of the DLMOs on days 1 and 4 (Day 1–Day 4) and statistically analyzed by using one-way ANOVA.

Two-way ANOVA for repeated measurements (Group and Day, Time and Group, or Time and Day) was used for sleep efficiency and sleep latency from the actigraphs, the OSA sleep inventory, and saliva and urine melatonin concentrations; in addition, for saliva melatonin concentrations, three-way ANOVA for repeated measurements (Group, Day, and Time) was also used. Differences in main effects were investigated using Bonferroni corrections.

The analyses were performed useing SPSS (Ver. 19, IBM, Tokyo, Japan), and a *P* value of <0.05 was considered to be statistically significant.

## Results

**Figure 2:**
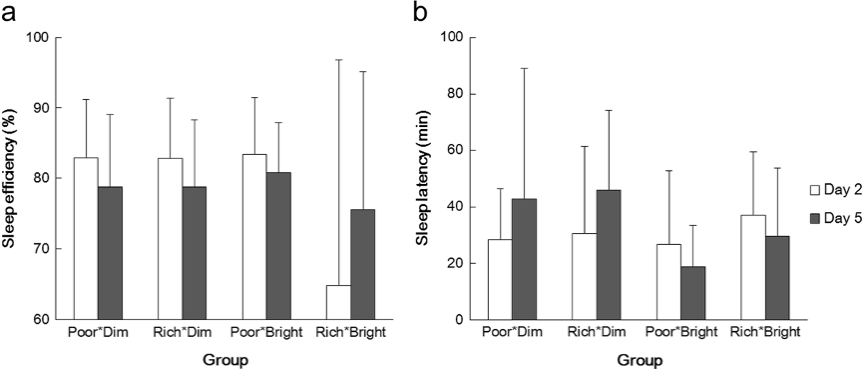
**Mean score (and SDs) for sleep efficiency (a) and latency (b) of the four groups on days 2 (white bars) and 5 (filled bars).**

**Figure 3:**
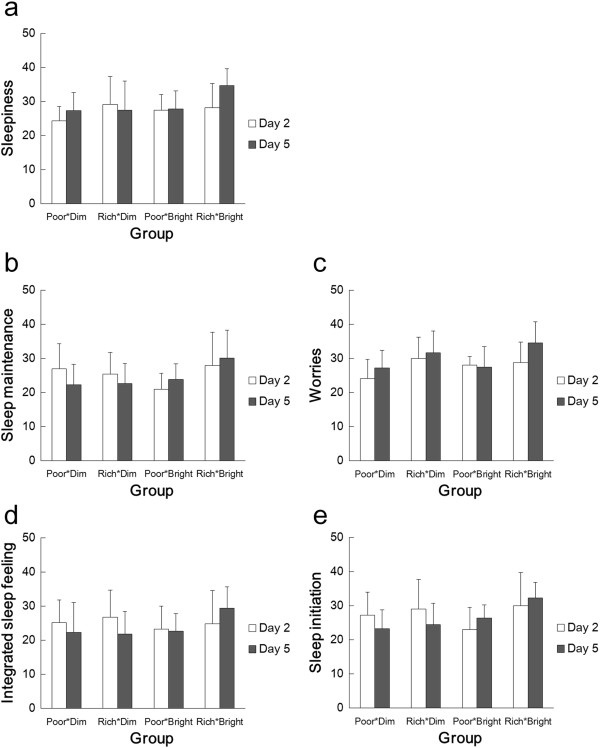
**Mean score (and SDs) for scores of the OSA sleep inventory in the four groups on days 2 (white bars) and 5 (filled bars). (a)** Sleepiness, **(b)** Sleep maintenance, **(c)** Worries, **(d)** Integrated sleep felling, and **(e)** Sleep initiation.

There were no significant differences (between days 2 and 5 and the four groups) or interactions (between Group and Day) with regard to sleep efficiency and sleep latency, as assessed from the actigraphs (two-way ANOVA, Group and Day; Figure 2). However, even though there were no significant differences between scores for the individual groups (multiple comparisons using the Bonferroni method), ANOVA indicated that there were statistically significant differences between the four groups overall in the scores for sleepiness (*P* <0.01), sleep maintenance (*P* <0.05), and worries (*P* <0.05) from the OSA sleep inventory (Group and Day; Figure 3). Main effects showed that sleepiness and worries scores differed significantly with Day (sleepiness, F (1, 33) = 6.0, *P* <0.05; worries, F (1, 33) = 10.9, *P* <0.01) when they were compared on the second and fifth days. The sleepiness score was 27.3 ± 1.0 (mean ± SE) on the second day and 29.3 ± 1.0 on the fifth day, and the worries score was 27.7 ± 0.9 on the second day and 30.2 ± 1.0 on the fifth day, which means that sleep quality was better (estimated as the result of sleepiness) and they were less worried on the fifth day compared to the second. Furthermore, although there were no significant differences between the scores on days 2 and 5, in Rich*Bright, the mean scores of sleepiness, sleep maintenance, worries, integrated sleep feeling, and sleep initiation on day 5 were higher than on day 2.

Regarding saliva melatonin concentration, there was a significant interaction between Group and Day (F (3, 29) = 6.2, *P* <0.01), but no interaction was found in relation to Time (three-way ANOVA; Group, Day, and Time). Saliva melatonin concentrations showed significant differences according to the time of the evening (F (6, 174) = 49.6, *P* <0.01).

**Figure 4:**
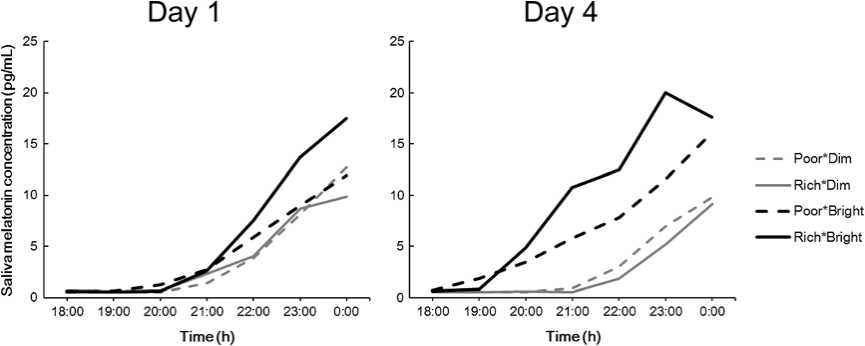
**Average saliva melatonin concentrations during the evenings of day 1 and day 4.**

**Figure 5:**
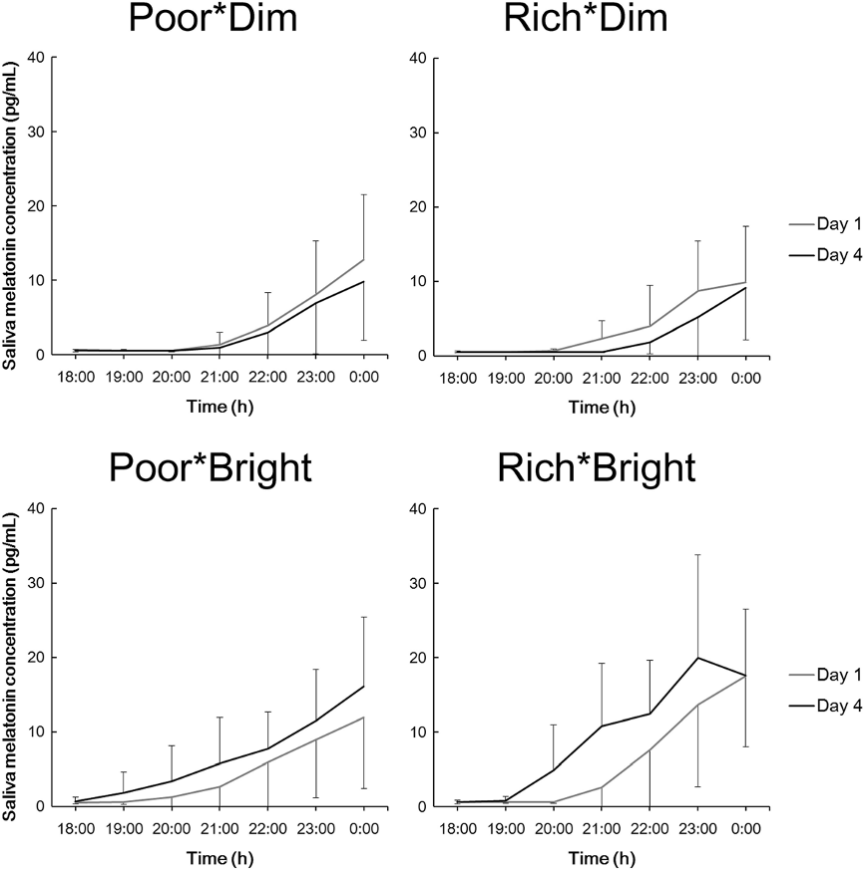
**Average saliva melatonin concentrations (and SDs) during the evenings of days 1 and 4 in each group.** Note that the data show the same as those in Figure 4.

In a further analysis of the effects of tryptophan and light condition upon saliva melatonin, concentrations during the evenings of days 1 and 4 were separately assessed by ANOVA for differences between Group and Time (Figure 4). On day 1, there were no significant differences between the four groups whereas, on day 4, the concentrations in the groups differed significantly (*P* <0.01). Multiple comparisons showed that, on day 4, there were significant differences between Poor*Dim and Rich*Bright (*P* <0.01) and Rich*Dim and Rich*Bright (*P* <0.01). Furthermore, saliva melatonin concentrations during the evenings of days 1 and 4 were compared by ANOVA for differences between Time and Day (Figure 5). There were significant differences between the concentrations on days 1 and 4 in the groups Poor*Dim (F (6, 54) = 5.0, *P* <0.05) and Rich*Bright (F (6, 36) = 2.9, *P* <0.05), but not in Rich*Dim and Poor*Bright. Compared with the first day, saliva melatonin secretion on the fourth day was lower in Poor*Dim but higher in Rich*Bright.

On days 1 and 4, the DLMO was considered for phase shifts of melatonin secretion. Table 3 shows the DLMOs of the four groups. There were significant differences between the shifts of DLMOs (Day 1–Day 4) in the four groups (one-way ANOVA, Group, *P* <0.01). Multiple comparisons showed that there were significant differences between Poor*Dim and Poor*Bright (*P* <0.01), Poor*Dim and Rich*Bright (*P* <0.01), Rich*Dim and Poor*Bright (*P* <0.01), and Rich*Dim and Rich*Bright (*P* <0.01). The DLMOs were significantly advanced by bright light exposure but not by a tryptophan-rich breakfast.

**Figure 6:**
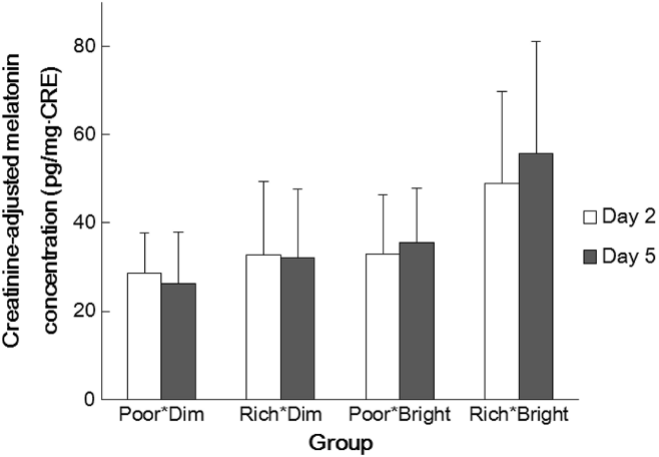
**Mean creatinine-adjusted melatonin concentration (and SDs) of the four groups in the rising sample taken on days 2 (white bars) and 5 (filled bars).**

Creatinine-adjusted melatonin in urine in the rising samples taken on days 2 and 5 are shown in Figure 6. In groups Poor*Dim and Rich*Dim, the mean values of the concentrations were lower on day 5, whereas, in Poor*Bright and Rich*Bright, they were higher on day 5. These results showed the same direction as saliva melatonin secretion. However, there were no statistically significant differences between the days, and no significant interaction between the Group and Day (two-way ANOVA).

**Table 3:** **DLMOs of the four groups on days 1 and 4**

Group	Number of subjects	DLMO* ^1^		
		Day 1	Day 4	Day 1–Day 4 (min)
Poor*Dim	8	21:53 (54)	22:12 (58)	−19 (13)
Rich*Dim	6	21:30 (60)	22:36 (47)	−66 (14)
Poor*Bright	7	21:23 (64)	19:57 (75)	86 (32)
Rich*Bright	7	21:38 (60)	20:07 (52)	90 (53)

*^1^Mean time of day and SDs are shown for DLMO on days 1 and 4.

## Discussion

The effects of a tryptophan-rich or tryptophan-poor breakfast and bright or dim light exposure during daytime upon sleep efficiency and latency (using actigraphy), five subjective aspects of sleep (from the OSA sleep inventory), and saliva and urine melatonins have been examined.

Although there were no significant group differences between saliva melatonin concentrations on the first day, significant differences were present on the fourth (Figure 4). The significant differences were between the Poor*Dim and Rich*Bright groups and between Rich*Dim and Rich*Bright groups (in both cases being higher in the Rich*Bright group). These results indicate that a tryptophan-rich breakfast and bright light exposure during daytime could promote saliva melatonin secretion at night; further, the observations that the Rich*Bright group had higher melatonin concentrations than the Rich*Dim group despite no significant differences between Poor*Dim and Rich*Dim nor Poor*Bright and Rich*Bright, suggests that bright light exposure in the daytime is an important contributor to raised melatonin levels in the evening, as has previously been found by others [19–22]. In addition, the DLMOs of saliva samples were significantly advanced by bright light exposure (Table 3). It is reported that bright light exposure (5,000 lx) in daytime (11:00–17:00 h) raised the following melatonin secretion at night and advanced its phase shift [19]; the results herein were in agreement with this report.

As shown in Figure 5, comparisons of saliva melatonin concentrations on the first and fourth days showed significant differences in groups Poor*Dim and Rich*Bright but no significant differences in groups Rich*Dim and Poor*Bright. The differences between days 1 and 4 were in the opposite direction in groups Poor*Dim and Rich*Bright; in the Poor*Dim group, melatonin secretion on the fourth day was lower than on the first day, whereas it was higher on the fourth day in Rich*Bright. It remains to be investigated if this change in the time-course of melatonin secretion observed in the evening continues until the following morning – that is, if there are phase advances/delays in the secretory profile and/or if there are changes in the total amount of melatonin secreted during the course of the whole night. However, in urine melatonin secretion, compared with the second day, the mean melatonin concentration on the fifth day was lower in Poor*Dim but higher in Rich*Bright (Figure 6). Although not statistically significant, these results show the same direction as found in saliva concentration (Figure 5). Therefore, the changes in melatonin secretion in saliva could indicate an increase of melatonin secretion and/or a phase shift of melatonin secretion. This point will be investigated by measuring saliva melatonin concentrations after midnight in a further study. Furthermore, only in groups Poor*Dim and Rich*Bright there were significant differences between saliva melatonin concentrations on days 1 and 4, and no significance was found in groups Rich*Dim and Poor*Bright (Figure 5). These results indicate that a combined effect of tryptophan intake and bright light exposure – an increase of salivary melatonin after a tryptophan-rich breakfast was augmented by bright light exposure during the daytime and vice versa – provides information that can be used to promote a healthy lifestyle.

Increased melatonin secretion raises the issue of changes in sleep. Although no significant differences were found for objective measures of sleep efficiency and latency, subjective feelings of sleepiness and worries were significantly improved on the fifth day compared to the second. In addition, only in Rich*Bright, all mean scores (sleepiness, sleep maintenance, worries, integrated sleep feeling, and sleep initiation) on day 5 were higher than on day 2. Although there were no significant differences between the scores on days 2 and 5, it is possible that tryptophan intake and bright light exposure improve subjective sleep quality. However, since there were no significant differences between the four groups, it is also possible that there was a general improvement in sleep over the course of the experiment, unrelated to the treatments. This improvement might result from the subjects becoming accustomed to the conditions in the experimental chamber. Furthermore, effects of light and tryptophan might depend upon the age and sensitivity of subjects [13, 23]. Since the subjects were young and healthy, the tryptophan and light effects might not be strong enough to cause clear sleep changes, although our bright condition (>5,000 lx) was bright enough to occur only rarely in most individuals’ daily lives. The generality of the present findings remains to be elucidated; for example, whether these results apply to females, older subjects, and non-Japanese populations whose basic diet is different.

It should also be considered why a tryptophan-rich breakfast and daytime exposure to bright light had no effects on objective (actigraphic) measures of sleep efficiency and latency. One reason might be due to limitations of the Actiwatches, which, although convenient for the subject, do not assess sleep architecture, they overestimate sleep latency, total sleep time, and sleep efficiency, and underestimate intermittent awakenings [24, 25]. Another reason might be the sample size used in this study; a larger sample size would help increase the accuracy of data analysis and the small group sizes might have led to the present negative results (a Type 2 error). In addition, Silber and Schmitt [23] suggested that, in healthy adults, tryptophan intake during the daytime had a relaxing and calming effect whereas taking it at night might have only a minimal effect on the subsequent sleep [23]. Therefore, the effects of the timing of tryptophan intake upon mood and sleep require further study.

The study design is also limited with regard to an absence of complete control of the food eaten at breakfast; although the TRP/LNAA ratio was controlled, as shown in Table 2, other nutrients and calorie intake differed. It is possible that these other factors affected the OSA scores and melatonin secretion. A stricter regulation of the subjects’ diet would be required to investigate these effects, even though complete control of the diet would be difficult to achieve. One way to investigate an effect of tryptophan independent of other nutrients is to administer pure tryptophan without food manipulation, as was done by Markus et al. [13, 14]. Their results showed that pure tryptophan and tryptophan-rich protein hydrolysate had greater effects on the plasma TRP/LNAA ratios and brain tryptophan availability, and thus caused mood and performance improvements. The effects of pure tryptophan or tryptophan-rich protein hydrolysate on melatonin secretion and sleep will be investigated in our next study.

## Conclusions

This study is the first to report quantitative effects of changed tryptophan intake at breakfast combined with daytime light exposure on melatonin secretion and sleep quality. Evening saliva melatonin secretion changed significantly and indicated that a tryptophan-rich breakfast and bright light exposure during the daytime promoted melatonin secretion at this time.

## Abbreviations

CV: Coefficients of variation
DLMO: Dim light melatonin onset
LoD: Limit of detection
LoQ: Limit of quantification
Poor*Bright: Tryptophan-poor breakfast and bright light environment
Poor*Dim: Tryptophan-poor breakfast and dim light environment
Rich*Bright: Tryptophan-rich breakfast and bright light environment
Rich*Dim: Tryptophan-rich breakfast and dim light environment
TRP/LNAA: Tryptophan/large neutral amino acids ratio.
